# Efficacy and Failure Patterns of Early SBRT to the Primary Tumor in Advanced EGFR-Mutation-Positive Lung Cancer with EFGR-TKI Treatment: A Prospective, Single Arm, Phase II Study

**DOI:** 10.3390/life12121954

**Published:** 2022-11-22

**Authors:** Yangyang Shi, Hailing Xu, William Y. Raynor, Jiapei Ding, Ling Lin, Chao Zhou, Wei Wang, Yinnan Meng, Xiaomai Wu, Xiaofeng Chen, Dongqing Lv, Haihua Yang

**Affiliations:** 1 Key Laboratory of Radiation Oncology of Taizhou, Radiation Oncology Institute of Enze Medical Health Academy, Affiliated Taizhou Hospital of Wenzhou Medical University, Taizhou 317000, China; 2 Department of Radiation Oncology, University of Arizona, Tucson, AZ 86721, USA; 3 Department of Pulmonary Medicine, Enze Hospital, Affiliated Taizhou Hospital of Wenzhou Medical University, Taizhou 317000, China; 4 Department of Radiology, Rutgers Robert Wood Johnson Medical School, New Brunswick, NJ 08901, USA; 5 Department of Radiation Oncology, Affiliated Taizhou Hospital of Wenzhou Medical University, Taizhou 317000, China; 6 Enze Medical Health Academy, Affiliated Taizhou Hospital of Wenzhou Medical University, Taizhou 317000, China; 7 Department of Radiation Oncology, Indiana University School of Medicine, Indianapolis, IN 46202, USA

**Keywords:** non-small cell lung cancers, stereotactic body radiation therapy, epidermal growth factor receptor mutation, tyrosine kinase inhibitor, phase II study

## Abstract

Early stereotactic body radiation therapy (SBRT) to the primary tumor combined with epidermal growth factor receptor tyrosine kinase inhibitor (EFGR-TKI) treatment may increase progression-free survival (PFS) by delaying resistance in patients with advanced EGFR-mutant non-small cell lung cancer (NSCLC). In this prospective, single arm, phase II study, patients with advanced NSCLC were treated with EGFR-TKI (icotinib 125 mg tid or gefitinib 250 mg qd) for one month followed by SBRT (40–60 Gy/5–8 F/5–10 d) to the primary tumor with concurrent EGFR-TKI until disease progression. The primary endpoint was PFS and the patterns of failure. Overall survival (OS) and adverse effects (AEs) were secondary endpoints. Overall, 41 advanced NSCLC patients with EGFR mutations received treatment with 24.42 months of median follow-up time. On average, SBRT was initiated 1.49 months after EGFR-TKI administration. Tumors were found to have an average shrinkage rate of 42.50%. Median PFS was 15.23 months (95% CI 13.10–17.36), while median OS was 27.57 months (95% CI 23.05–32.09). Thirty-three patients were found to have disease progression, of which new site failure (NF) (22 patients, 66.66%) was the most common pattern, followed by original site failure (OF) (7 patients, 21.21%) and simultaneous OF/NF (ONF) (4 patients, 12.12%). There were no Aes equal to or greater than grade 3, with the most frequent AE being radiation pneumonitis. Therefore, administering therapy targeted at the primary tumor using early SBRT after EGFR-TKI initiation is a new potentially safe and effective approach to treat EGFR-mutant advanced NSCLC.

## 1. Introduction

As one of the most common cancers worldwide, lung cancer makes up approximately a quarter of all cancer deaths [1]. A previous cohort study estimated that 55.9% of advanced lung adenocarcinoma patients in eastern China have activating epidermal growth factor receptor (EGFR) mutations [2]. Tyrosine kinase inhibitors (TKI), which target EGFR, were the standard treatment for metastatic non-small cell lung cancer (NSCLC) harboring EGFR mutations [3]. Compared to traditional chemotherapy, first-generation EGFR-TKI treatment can significantly improve the survival rate, progression-free survival (PFS), and overall survival (OS), allowing for a 5-year survival rate of approximately 20%, 9–13 months of PFS, and 19–30 months of OS [4,5].

However, acquired targeted resistance was encountered, the mechanisms of which including those dependent on the EGFR signaling pathway, those independent of the EGFR signaling pathway, and small cell transformation [6]. The most common resistance mechanism was found to be the T790M mutation, with a 36.51% (69/189) plasma detected rate reported previously [7].

Several studies reported that initial progression of TKI-treated lung cancer occurred predominantly at original disease sites [8,9]. Furthermore, the size of the primary lung tumor was strongly associated with the incidence of failure at the original site. EGFR-TKI was found to be radiosensitive both in vivo and in vitro. The EGFR-TKI afatinib combined with radiotherapy significantly increases the anti-tumor effect of radiation in PC-9-GR cells harboring acquired T790M [10]. 

Stereotactic body radiation therapy (SBRT), a novel method of radiotherapy, has superior precision, increased potency per fraction for tumors, as well as decreased damage to normal tissue compared to conventional radiation treatment. Recently, SBRT has demonstrated a tremendous role in the treatment of lung cancer [11,12,13,14]. Thus, many researchers hypothesized that SBRT for residual disease could delay subsequent metastatic reseeding, thereby eliminating tumor internal heterogeneity. For instance, in a phase II study by Kong et al., it was found during midtreatment positron emission tomography (PET) imaging that there was a favorable local regional tumor control for NSCLC receiving radiation with concurrent chemotherapy [15]. In metastatic disease, studies have shown the potential role of SBRT in achieving high local control in patients with oligo-progressive NSCLC, and there has been increasing interest in SBRT for oligo-metastatic NSCLC, including cases which involve the lungs, brain, liver, spine, and adrenal glands [16,17,18]. Similar results were gained in oligo-progressive and oligo-metastatic NSCLC with EGFR mutations; for instance, the randomized SINDAS trial, which found that preemptive RT before the occurrence of oligo-progression improved OS and PFS in these populations [19], although the definitions of oligo-metastatic and oligo-progression varied among studies. A phase II prospective study demonstrated that radiotherapy to all intrathoracic sites within 2 weeks from the initiation of EGFR-TKI treatment obtained 13.0 months of PFS with well-tolerated side effects [20]. Furthermore, it was also found that the addition of upfront local therapy with RT followed by TKI treatment statistically improved PFS and OS for EGFR-mutated NSCLC [21].

Therefore, we hypothesized that the early SBRT to the primary tumor shortly after initiation of treatment with EGFR-TKI therapy could also prevent progression and prolong PFS in EGFR-mutation-positive NSCLC by delaying the development of targeted resistance.

## 2. Materials and Methods

### 2.1. Study Design and Participants

A prospective, single arm, phase II trial (ChiCTR-OIN-17013920) was conducted. Approval was obtained from the institutional ethics review board. All participants provided written informed consent.

The key eligibility criteria were as follows: age ≥18 years and ≤85 years; Eastern Cooperative Oncology Group (ECOG) performance status (PS) of 0 or 1; histopathologically or cytologically proven non-small cell lung cancer (NSCLC). The patient selection process included review of a complete medical history, physical examination, fiberoptic bronchoscope or CT-guided needle biopsy results, pathologic diagnosis, complete blood counts, liver and renal function tests, cross-sectional imaging of the chest, abdomen, head, and neck, including by CT, MRI, ultrasound, and PET/CT. Initial treatment of stage IV, or advanced IIIB, or IIIC who refused concurrent radiochemotherapy and other treatments according to TNM version 8; harboring EGFR mutation (Exon 21 L858R or Exon 19 deletion) by ARMS test; brain metastases would be eligible if they were asymptomatic or completed treatment using 25 Gy with 10 fractions whole brain radiotherapy (WBRT) ≥14 days before starting study treatment [22]; adequate organ function. The study was designed to enroll 50 patients to detect 6 months’ extension of PFS.

### 2.2. Treatment 

All patients were administered a first-generation EGFR-TKI (icotinib 125 mg tid or gefitinib 250 mg qd, orally). Participants who achieved partial response (PR) or stable disease (SD) after one month of EGFR-TKI treatment according to Response Evaluation Criteria in Solid Tumors version 1.1 (RECIST v1.1) received SBRT with 40–60 Gy/5–8 Fraction/5–10 days to the primary tumor while continuing targeted therapy. Lung contouring was done according to RTOG 0915. GTV was tumor-contoured, utilizing CT lung windows, and also verified on mediastinal windows to avoid inclusion of adjacent vessels. ITV was generated to account for tumor motion from 4D-CT data set contouring GTV in each phase of the respiratory cycle. PTV was ITV + 0.5 cm uniform expansion; this was done in accordance with our previous study by Tang et al. [23].

### 2.3. Assessments 

Tumor imaging was performed at baseline, after one month, and every 3 months thereafter. According to RECIST v1.1, PR was defined as a reduction of ≥30% in the longest diameter of the target tumors measured by computed tomography (CT) compared to the baseline, and progressive disease (PD) was defined as an increase of ≥20% in the maximum diameter of the target tumors compared with these recorded after treatment initiation or the occurrence of one or more new tumors. SD was defined as the intermediate between PR and PD. Tumor shrinkage rate was evaluated according to the response of primary lesion by RECIST v1.1.

As for failure pattern models, it was classified as original site failure (OF), including progression in initial primary or metastatic lesions, or new site failure (NF), respectively. Simultaneous OF/NF was labeled as ONF. 

Adverse effects (AEs) were recorded throughout treatment and for 30 days thereafter (90 days for serious AEs), including any occurrences of nausea, vomiting, diarrhea, rash, paronychia, transaminitis, increased creatinine, neutropenia, radiation pneumonia, or radiation esophagitis, and graded according to the National Cancer Institute Common Terminology Criteria for Adverse Events version 5.0 (CTCAE v5.0). Survival was assessed every 4 weeks during follow-up.

### 2.4. Endpoints

The primary endpoint was PFS, which was defined as the time from EGFR-TKI treatment to disease progression, and the patterns of failure. Secondary endpoints were AEs and OS, which was defined as the time from EGFR-TKI treatment to death. These results are compared to existing literature on EGFR-TKI inhibitor alone in terms of failure, AE, OS, and PFS.

### 2.5. Statistical Analysis

Given an expected median PFS of 16 months, the number of patients was calculated in order to provide a statistical power of 80% to confirm the superiority of the lower confidence boundary of the observed median PFS compared with the threshold median PFS of 10 months. A two-sided, one-sample logrank test calculated from a sample of 54 subjects achieves 80.6% power at a 0.050 significance level to detect a hazard ratio of 0.625 when the median PFS of the historic control group is 1.00. The probability that a subject experiences an event during the study is 0.6588. 

All statistical assessments were performed on the statistical package for the social sciences (SPSS) version 20.0 (SPSS Inc., Chicago, IL, USA). For PFS, OS, and their stratified analysis, event-time distributions were estimated by the Kaplan–Meier method. Cox proportional hazards models were used to assess the contribution of each potential prognostic factor for survival analysis, including the hazard ratio (HR) and 95% confidence interval (CIs). Because M stage, pathological stage, and the number of metastases were all interdependent, the number of metastases was chosen as the only one to be included in the multivariate analysis. All *p* values were two-tailed. A *p* value ≤ 0.05 was considered statistically significant. The results of the patients receiving SBRT after EGFR-TKI were followed for PFS, OS, SE, and patterns of failure.

## 3. Results

### 3.1. Patients

From September 2016 to November 2021, 41 patients were enrolled in total. The study was closed after 41 patients enrolled due to the new availability of more effective third-generation EGFR-TKI agents. Baseline characteristics are shown in Table 1.

**Figure 1:**
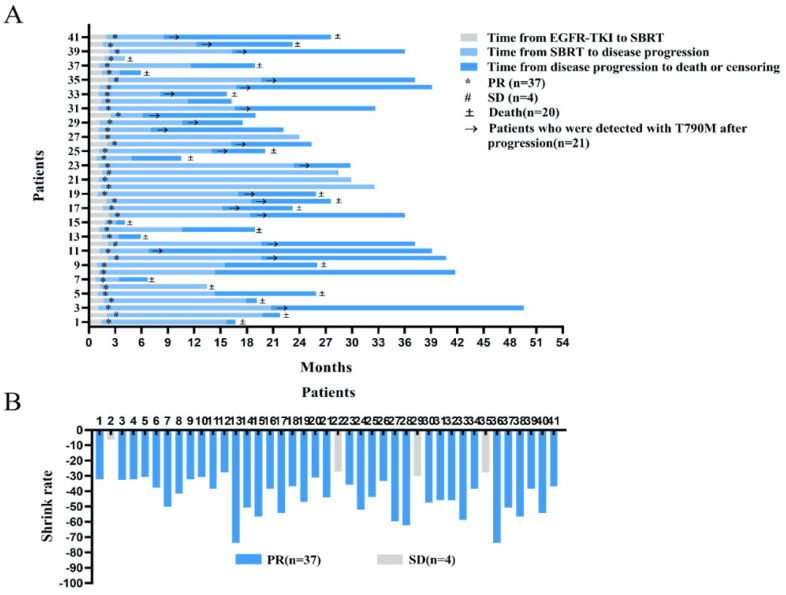
Follow−up time of patients. (**A**) Changes of treatment in different times; (**B**) distribution of objective response in patients.

There were 37 patients with partial response (PR) and 4 patients with stable disease (SD) after one month of treatment with EGFR-TKI (Figure 1A).

A mean time interval of 1.49 months (0.73–2.47 months) occurred between initiation of EGFR-TKI and SBRT. 

### 3.2. Efficacy

Overall, 41 advanced NSCLC patients with EGFR mutations received treatment with 24.42 months of median follow-up time. On average, tumors were found to have a shrinkage rate of 42.50% (Figure 1B). 

**Figure 2:**
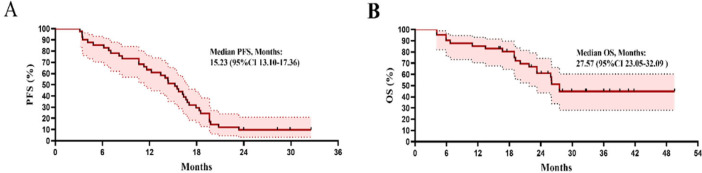
Survival analysis of patients in the whole group. (**A**) Median PFS; (**B**) median OS.

**Figure 3:**
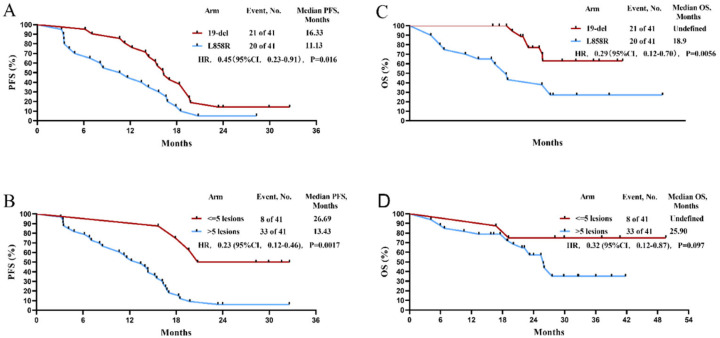
Survival analysis of patients in subgroups. (**A**) PFS in EGFR mutation subtype group; (**B**) PFS in the number of metastases subtype group; (**C**) OS in EGFR mutation subtype group; (**D**) OS in the number of metastases subtype group.

As shown in Figure 2A, the median PFS was 15.23 months (95% CI 13.10–17.36). The PFS of subgroups in number of metastases >5 vs. <5 and EGFR mutation L858R vs. 19-del were 11.13 months vs. 16.33 months (HR 0.45, 95% CI (0.23–0.91), *p* = 0.016) and 13.43 months vs. 26.69 months (HR 0.23, 95% CI (0.12–0.46), *p* = 0.0017), respectively (Figure 3). The PFS benefit of adding SBRT after EGFR-TKI was observed in subgroups of T1–2, number of metastases (≤5 lesions), 19-del mutation in Univariate analysis and sites of metastasis (>5 lesions vs. ≤5 lesions, HR 4.3, 95% CI (1.3–15.0), *p* = 0.02), type of EGFR mutation (L858R vs. 19-del, HR 2.8, 95% CI (1.4–5.6), *p* = 0.005) as determined by multivariate analysis (Table S1), respectively.

As shown in Figure 2B, the median OS was 27.57 months (95% CI 23.05–32.09). 

The OS of subgroups in number of metastases >5 vs. <5 and EGFR mutation L858R vs. 19-del were 18.90 months vs. Undefined (HR 0.32, 95% CI (0.12–0.87), *p* = 0.097) and 25.90 months vs. Undefined months (HR 0.29, 95% CI (0.12–0.70), *p* = 0.0056), respectively (Figure 3C,D). The higher OS benefit of SBRT to TGFR-TKI was observed only in subgroups of 19-del mutation by univariate analysis (L858R vs. 19-del, HR 3.5, 95% CI (1.4–9.2) and demonstrated by multivariate analysis (L858R vs. 19-del, HR 3.5, 95% CI (1.4–9.2) (Table S2).

### 3.3. Safety

The main AEs are outlined in Table 2. Radiation pneumonitis (85.37%), transaminitis (51.22%), and rash (39.02%) were the top three most common side effects. The most common Grade 2 AEs was transaminitis (31.71%). There were no AEs of Grade 3 or above.

### 3.4. Patterns of Failure

As listed in Table 3, of 33 patients who progressed, 7 (21.21%) had OF, 22 (66.66%) had NF, and 4 (12.12%) had ONF. The lung was the most common site of initial progression.

## 4. Discussion

This was the first study to explore the efficacy of early SBRT to the primary tumor in advanced cases of EGFR-mutant NSCLC after first-generation EGFR-TKI and to determine the patterns of failure of this combination therapy. The primary endpoints were PFS and the patterns of failure. OS and adverse effects (AEs) were second endpoints. Encouragingly, the median PFS of SBRT combined with EGFR-TKI treatment in advanced NSCLC patients was 15.23 months without serious AEs. The incidence of radiation pneumonitis, the most common AE, was 85.7%, and the majority were Grade 1. As for the patterns of failure in the relapsing 37 patients, failure mainly occurred at new metastatic sites. Overall OS was favorable at 27.57 months. Local SBRT to the primary tumor in the early phase, at approximately the first month in our study, seemed to have good local control, with the possibility of eliminating tumor heterogeneity and thereby delaying targeted resistance in advanced EGFR-mutation-positive NSCLC treated with EGFR-TKI. 

Consistent with previous studies, there was a significant difference in PFS and OS when patients with the 19-del mutation were compared with those who had the L858R mutation. In a study by Tang et al., patients with the 19-del mutation were more likely to benefit from combination therapy consisting of SBRT and EGFR-TKI [24]. In the NEJ026 study, bevacizumab plus erlotinib combination therapy could also improve PFS compared with erlotinib alone in patients with EGFR-mutation-positive non-squamous NSCLC, especially for patients with the L858R mutation [25]. However, bevacizumab plus erlotinib did not significantly affect OS, and no difference was observed in mutation subtype analysis. Similar results were found in the combination of EGFT-TKI and chemotherapy, but mutation subtype analysis was not performed. 

In the subgroup analysis, the number of metastases (≤5 lesions) was only a predictor for PFS, rather than OS, in patients receiving SBRT to the primary tumor combined with EGFR-TKI, but a trend of improved OS was observed. As previously demonstrated, consolidative therapy confers better survival benefit than maintenance therapy alone [17]. Numerous prospective and retrospective studies indicated that local radiotherapy combined with continuation of TKI therapy limited the progression, noted as oligo-progression, of naive EGFR mutation with TKI [26,27]. For instance, in a recent study by Wang et al., it was found that local therapy with RT followed by TKI treatment statistically improved OFS and OS for EGFR-mutated NSCLC [21]. Xu et al. also proposed local treatment for local progression models in oligo-metastasis of EGFR-mutant NSCLC patients with first-line EGFR-TKI before progression [28], although the number of metastases varies between studies. Consistent with our results, in patients with five or fewer metastases, SBRT prolonged PFS by 26.69 months, and an increasing trend in OS was observed. Although most patients in this group were stage IIIB, it was also in line with a multi-center and retrospective study of unresectable advanced NSCLC with EGFR mutation, which revealed that radiotherapy combined with EGFR-RKI could prolong PFS by 21.6 months and OS by 67.4 months when compared with the chemoradiotherapy group and the EGFR-TKI alone group [29]. 

The time of intervention with local treatment in oligo-metastatic EGFR-mutation-positive NSCLC remains controversial. As for patients with limited brain metastases, the sequence of EGFR-TKI and local treatment (for example: SRS, SBRT, or surgery) depends on central nervous system symptomatology. As for lung sites, many proposals suggest that introducing local therapy at or near the time of TKI initiation could hold the potential to reduce initial accumulation of malignant clones, decreasing the risk of subsequent metastasis and reducing the injury to normal tissue due to the reduction of lesion burden [30]. A retrospective study with 105 subjects indicated that considerable shrinkage from TKI therapy occurs in the first 2 months after TKI initiation [24]; local therapy can therefore be adopted after this timepoint and before disease progression, especially for EGFR-mutation-positive patients. In a study by Wu et al., the median response time after TKI treatment was 7.4 weeks [31]. In the present study, there were 33 PR and 4 SD. The median response time was 1.49 (0.73–2.47) months, similar to that of previous studies. Another study by Wei et al. showed that preemptive RT to the primary lung tumor before occurrence of oligo-metastasis significantly improved PFS, also suggesting the benefit on survival with early intervention of RT [19]. However, future prospective studies of RT in the early phase of TKI initial treatment in NSCLC are imperative, as it remains unknown whether or not RT before or after chemotherapy provides better survival outcomes in patients with EGFR-mutant NSCLC.

In metastases resulting from EGFR-mutation-positive NSCLC, reports of RT mainly focus on alleviating symptoms and multi-brain metastasis. Several studies demonstrated that WBRT with EGFR-TKI would not improve OS, with greater impaired cognitive function and decreased quality of life. As for lung tumors, Zheng et al. demonstrated that concurrent EGFR-TKI and radiotherapy within 2 weeks of targeted therapy as the first-line treatment for advanced NSCLC harboring the EGFR mutation showed a long-term control of primary lung lesion and acceptable serious AEs, with 13.0 months of PFS and a 57.1% 1-year PFS rate [20]. Conforming to our research, patients with more than 5 metastases were found to have 13.43 months of PFS and 25.9 months of OS, although SBRT was administered approximately 1 month after TKI and only for the primary tumor in the lung. A significantly lower rate of progression was observed at the site of the primary tumor, which was consistent with the study by Al-Halabi et al. [9]. However, some studies demonstrated that all forms of RT could obtain PFS and OS benefit, rather than part RT or RT alone [28]. The role of RT in combination therapy is still under controversy, and there are no consensus guidelines for the use of RT in EGFR-mutant NSCLC [32].

The mechanism underlying the efficacy of concomitant SBRT and EGFR-TKI remains unclear. Notably, of progression in 37 patients, a total of 24/37 (64.86%) were under second gene detection, and the T790M mutation was detected in 21/37 patients (56.76%), including 14 patients (66.67%) with the initial 19-del mutation and 7 patients (33.33%) with the L858R mutation. It was consistent with the occurrence rate of T790M after targeted therapy and in line with the phenomenon of the 19-del mutation developing from T790M rather than L858R [7,33,34].

In terms of further generations of EGFR-TKI therapy, the second generation of EGFR-TKI in clinical application was limited, although it could prolong PFS by 11 to 14.7 months, owing to its relatively higher potency. Outstanding performance was demonstrated by the third-generation EGFR-TKI agent osimertinib, which is the first-line treatment for EGFR-mutation-positive NSCLC, especially for patients with the T790M mutation and brain metastasis [35]. The FLAURA trial suggests improvement in PFS and OS with osimertinib compared with gefitinib or erlotinib in EGFR mutant NSCLC [21]. The FLAURA trial had a PFS rate of 38.6 months vs. first-generation TKI of 31.8 months; however, this study had already started by the time the FLAURA results were published, resulting in unfortunate timing. Guo et al. analyzed the pattern of recurrence, finding 50%, 22%, and 28% for OF, NF, and ONF, respectively, in metastatic EGFR-mutation-positive NSCLC treated with simertinib [36]. The authors hypothesized that consolidative SBRT to all residual disease sites as an addition to EGFR-TKI therapy holds promise for delaying disease progression and even for improving OS.

With the advent of immunotherapy, the 5-year survival of NSCLC patients was approximately 30%, which was a victory over conventional chemotherapy. However, immunotherapy as a first-line therapy was abandoned in advanced NSCLC harboring the EGFR mutation in many clinical trials [37], despite a few positive results in IMpower15030 and ATLANTIC31. Regardless, immunotherapy was used in advanced EGFR-mutation-positive NSCLC after EGFR-TKI resistance developed in clinical trials, and its outcomes could be anticipated, given the theory that immune escape mediated by upregulation of PD-L1 was one of the targeted resistance mechanisms in vivo and in vitro [38]. 

To our knowledge, this was the first study to evaluate the effectiveness and patterns of failures in concurrent early-phase SBRT to the primary tumor after first-generation EGFR-TKI treatment in EGFR-mutant NSCLC. However, there are several limitations in our study. First, a limited number of patients were included, and no control group receiving EGFR-TKI alone was available as per a phase II trial. A larger, multi-center, and prospective phase III study is greatly needed. Secondly, resistance mechanisms underlying this regimen have not been explored, which may guide further treatment development. 

## 5. Conclusions

In summary, early-phase SBRT for the primary lung tumor combined with EGFR-TKI followed by RT may be an alternative choice for advanced NSCLC harboring the EGFR mutation, which has the potential to alter the course of disease progression and to delay targeted resistance, as well as increasing OS without serious AEs. The lung was the most common site of initial progression. It remains to be determined whether SBRT before or after chemotherapy is more beneficial for survival in EGFR-mutant NSCLC. The role of SBRT during treatment of EGFR-mutant NSCLC with osimertinib also warrants further consideration.

## Figures and Tables

**Table 1:** Patient characteristics (*n* = 41).

Characteristics		Number of Subjects
Age	Median, range (years)	66 (46–75)
	>66	23
	≤66	18
Sex	Female	18
	Male	23
Smoke history	Yes	16
	No	25
ECOG PS	0	15
	1	26
Mutation type	L858R	20
	19-del	21
T	T1–2	13
	T3–4	28
N	N0–1	9
	N2–3	32
M	0	7
	1	34
Lesions	1–5	1
	>5	33
Stage	III	7
	IV	34
Initial metastasis	Lung	20
	Brain	6
	Bone	21
	Liver	4
	Adrenal glands	1
	Regional lymph nodes	35
	Distant lymph nodes	1
	Others	15

**Table 2:** Main adverse effects.

	Grade 1	Grade 2	Grade 3	Grade 4	Grade 5	All (%)
Radiation pneumonitis	27	8	0	0	0	35 (85.37)
Radiation esophagitis	0	0	0	0	0	0 (0.00)
Transaminase increased	8	13	0	0	0	21 (51.22)
Renal insufficiency	6	0	0	0	0	6 (14.63)
Rash	14	2	0	0	0	16 (39.02)
Diarrhea	4	0	0	0	0	4 (9.76)
Paronychia	1	1	0	0	0	2 (4.88)
Hypokalemia	2	0	0	0	0	2 (4.88)
Nausea and vomiting	1	0	0	0	0	1 (2.44)

**Table 3:** Patterns of initial failure.

Sites of Initial Failure	All Patients (*n* = 33)		Patients with OF (*n* = 7)		Patients with NF (*n* = 22)		Patients with ONF (*n* = 4)	
	*n*	%	*n*	%	*n*	%	*n*	%
Brain	11	33.33	0	0	10	45.45	1	25
Bone	12	36.36	0	0	9	40.90	3	75
Liver	2	6.06	0	0	2	9.09	0	0
Lung	19	57.58	7	100	8	36.36	4	100
Regional lymph nodes	3	9.09	0	0	0	0	3	75
Others	4	12.12	0	0	1	4.55	3	75

OF: original site failure; NF: appearance of new lesions as distant site failure; ONF: Simultaneous OF/NF.

## Data Availability

The original contributions presented in the study are included in the article/Supplementary Materials, further inquiries can be directed to the corresponding authors.

## Supplementary Materials

The following are available online at https://www.mdpi.com/article/10.3390/life12121954/s1, Table S1: Univariate and multivariate analysis of covariables associated with PFS, Table S2: Univariate and multivariate analysis of covariables associated with OS.
