# Supplementary material for: Efficacy and Failure Patterns of Early SBRT to the Primary Tumor in Advanced EGFR-Mutation-Positive Lung Cancer with EFGR-TKI Treatment: A Prospective, Single Arm, Phase II Study
Source: Life (Basel). 2022 Nov 22;12(12):1954. doi: 10.3390/life12121954 (PMC9783042; doi:10.3390/life12121954)
Supplement: Supplementary file 1 [file life-12-01954-s001.zip › life-1988897-supplementary.pdf]

Table S1. Univariate and multivariate analysis of covariables associated with PFS.

	Univariate analysis			Multivariate analysis		
	HR	95%CI	P value	HR	95%CI	P value
Age, year (≦66 vs >66)	1.3	0.7-2.6	0.40			
Sex (Female vs Male)	1.0	0.5-1.9	0.90			
Smoke (No vs Yes)	1.1	0.5-2.2	0.80			
ECOG PS (0 vs1)	0.9	0.4-1.7	0.70			
T (T1-2 vs T3-4)	0.4	0.2-0.8	0.02	0.8	0.3-1.9	0.60
N (N0-1 vs N2-3)	1.0	0.5-2.0	0.90			
No. of metastasis (>5 vs 0-5 lesions)	5.8	2.0-16.8	0.001	4.3	1.3-15.0	0.02
EGFR mutation (L858R vs 19-del)	2.1	1.0-4.0	0.03	2.8	1.4-5.6	0.005
Response before radiotherapy (SD vs PR)	12.1	1.6-90.7	0.02	5.2	0.6-42.5	0.10

HR: hazard ratio; CI: confidence interval.

Table S2. Univariate and multivariate analysis of covariables associated with OS.

Independent Variable	Univariate analysis			Multivariate analysis		
	HR	95%CI	P value	HR	95%CI	P value
Age, year (≦66 vs >66)	1.3	0.6-2.8	0.5			
Sex (Female vs Male)	1.0	0.5-2.2	0.9			
Smoke (No vs Yes)	0.8	0.4-1.7	0.5			
ECOG PS (0 vs1)	1.2	0.6-2.7	0.6			
T (T1-2 vs T3-4)	0.5	0.2-1.2	0.1			
N (N0-1 vs N2-3)	1.3	0.5-3.3	0.6			
Number of metastasis(≤5 lesions vs >5 lesions)	3.2	0.7-13.8	0.1	0.3	0.7-16.7	0.12
EGFR mutation (L858R vs 19-del)	3.5	1.4-9.2	0.01	3.5	1.4-9.2	0.01
Response before radiotherapy (SD vs PR)	4.8	0.6-35.6	0.1			

HR: hazard ratio; CI: confidence interval.
